# Enabling Efficient Oxygen Reduction Reaction with Pt Single Atoms on Carbide: A Phosphorus-Doped Mo_2_C Interface Strategy

**DOI:** 10.1021/acs.nanolett.5c04201

**Published:** 2025-10-29

**Authors:** Changwei Shi, Xingmao Jiang, Xueqiang Qi, Congcong Xing, Xiaolei Fan, Zhuo Chen, Xiang Wang, Andreu Cabot

**Affiliations:** † School of Chemical Engineering and Pharmacy, Hubei Key Laboratory of Novel Reactor and Green Chemical Technology, 34756 Wuhan Institute of Technology, Wuhan 430205, China; ‡ State Key Laboratory of Green and Efficient Development of Phosphorus Resources, 34756 Wuhan Institute of Technology, Wuhan 430205, China; § Catalonia Institute for Energy Research (IREC), Sant Adrià de Besòs, 08930 Barcelona, Spain; ⊥ School of Chemistry and Chemical Engineering, Chongqing University, Chongqing 400044, China; ∥ Wenzhou Key Laboratory of Novel Optoelectronic and Nano Materials, Institute of Wenzhou, Zhejiang University, Wenzhou 325006, China; ¢ Department of Materials Science and Engineering, Zhejiang University, Hangzhou 310027, China; ○ Department of Chemical Engineering, School of Engineering, 5292 The University of Manchester, Oxford Road, Manchester M13 9PL, United Kingdom; △ 12565 State Key Laboratory of Advanced Technology for Materials Synthesis and Processing Wuhan University of Technology, Wuhan 430070, P. R. China; ¶ ICREA Pg. Lluis Companys, 08010 Barcelona, Catalonia, Spain

**Keywords:** single atoms, phosphorus doping, molybdenum carbide, heterostructure, oxygen reduction reaction, zinc−air battery

## Abstract

Developing efficient and cost-effective oxygen reduction reaction (ORR) catalysts is a critical process in electrochemical energy conversion technologies. Here, we report a new heterostructured electrocatalyst composed of phosphorus-doped Mo_2_C coupled with atomically dispersed Pt sites (Pt/P-Mo_2_C). This is realized through a confined polymerization approach using heteropolyacid–pyrrole complexes and subsequent covalent anchoring. Phosphorus doping plays a crucial role in enhancing the interfacial electron density and enabling strong electronic interactions with Pt atoms. The results showed that the interfacial electronic structure of Pt is significantly modulated, with a downshifted d-band center that optimizes the adsorption/desorption energetics of ORR intermediates. As a result, Pt/P-Mo_2_C demonstrates outstanding ORR activity in alkaline media, achieving a half-wave potential (*E*_1/2_) of 0.91 V along with excellent stability. This work presents a generic strategy for integrating single-atom noble metals with carbide supports and highlights the role of interfacial electron engineering in the design of next-generation ORR electrocatalysts.

## Graphical abstract

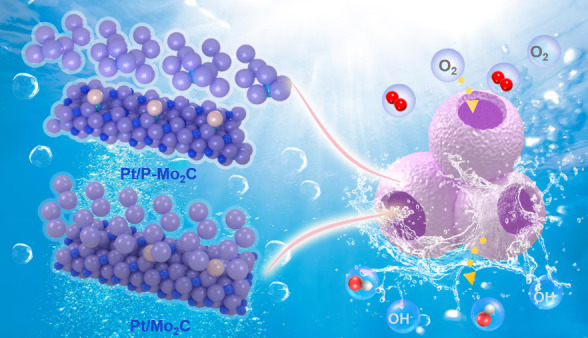

The oxygen reduction reaction (ORR) is a key half-reaction for sustainable energy and electrochemical conversion systems, especially in metal–air batteries and fuel cells.^1–3^ The challenge of ORR lies in developing electrocatalysts that are not only highly active but also durable and cost-effective.^4–6^ Platinum (Pt)-based catalysts remain the gold standard for ORR due to their superior activity, yet their high cost, scarcity, and poor atom utilization underscore the need for innovative catalyst design strategies that reduce Pt loading without compromising performance.^7,8^

One of the most promising approaches in this regard is the development of single-atom catalysts (SACs). By atomically dispersing Pt atoms on a support, SACs ensure maximum utilization efficiency, as every atom is exposed and potentially catalytically active.^9,10^ Moreover, the unique electronic properties and unsaturated coordination environment of single-atom Pt species can lead to altered adsorption energies of reaction intermediates, often resulting in enhanced catalytic performance.^8^ Nonetheless, the stabilization of isolated Pt atoms under electrochemical conditions remains a central challenge due to their inherent tendency to migrate and agglomerate into less active clusters or nanoparticles.^11^

The design of an effective SAC thus critically depends on the choice of support material, which must anchor Pt atoms securely while also participating synergistically in the catalytic process.^12^ Transition metal carbides (TMCs), particularly molybdenum carbide (Mo_2_C), have emerged as promising support materials for SACs.^13,14^ Mo_2_C exhibits a combination of metallic conductivity, chemical robustness, and catalytic behavior reminiscent of those of noble metals. Its unique d-band structure facilitates favorable interaction with adsorbates, making it a compelling candidate for ORR electrocatalysis.^15,16^ Moreover, Mo_2_C can promote charge transfer to or from the anchored metal atoms, tuning their electronic properties to enhance catalytic activity.^13,17,18^

Despite these advantages, pristine Mo_2_C still has limitations. Its surface can be relatively inert, and it may lack sufficient anchoring sites for the strong stabilization of single atoms.^19,20^ To address these issues, heteroatom doping has been widely explored as an effective strategy to modulate the surface chemistry, electronic structure, and defect density of catalyst supports.^21,22^ Among various dopants, phosphorus (P) stands out due to its unique ability to tune both the geometric and the electronic structure of the host material. Phosphorus doping introduces negatively charged P sites, which can modify the local electron density, create new coordination environments, and act as efficient anchoring sites for metal atoms.^23^ Additionally, P-doping can improve the conductivity and stability by suppressing surface oxidation and enhancing hydrophilicity. When phosphorus is incorporated into Mo_2_C, the resulting P–Mo_2_C support not only exhibits enhanced intrinsic ORR activity but also provides a more favorable environment for stabilizing Pt single atoms.^24^ The strong interaction between Pt and P-modified Mo and C sites can lead to optimized charge distribution, facilitating oxygen adsorption and activation while minimizing Pt atom migration.^25,26^ Furthermore, P-doping can lower the d-band center of the Mo_2_C support, fine-tuning the binding strength of ORR intermediates such as O_2_*, OOH*, O*, and OH*. Achieving this delicate balance is essential for maximizing the reaction rate and minimizing the overpotential in the ORR.

Moreover, the nanostructuring of the support plays a pivotal role in the catalyst performance. Molybdenum carbide nanospheres offer a well-defined, high-surface-area morphology that enhances the dispersion of active sites and facilitates mass transport.^27^ The uniform spherical structure reduces diffusion barriers and enhances contact with the electrolyte, while also enabling better control over surface doping and metal atom anchoring.^28^ When these nanospheres are doped with phosphorus and decorated with single Pt atoms, the resulting catalyst system combines atomic-level precision and mesoscopic structural advantages.

Herein, we used heteropolyacid pyrrole polymer nanospheres (PMo_12_-PPy NSs) as precursors including phosphorus and molybdenum, followed by simple wet chemical impregnation and thermal treatment to obtain single Pt atoms anchored onto phosphorus-doped molybdenum carbide nanospheres (Pt/P-Mo_2_C). Electrochemical measurements demonstrate that the Pt/P-Mo_2_C exhibit superior ORR activity in alkaline media compared to commercial Pt/C and Pt/Mo_2_C. Density functional theory (DFT) calculations reveal that P-doping modulates the electronic properties of the Pt–Mo_2_C interface, optimizing the binding strength of oxygenated species and reducing the overall energy barrier for the rate-determining step. Additionally, Pt/P-Mo_2_C is also employed to assemble zinc–air batteries (ZABs) with RuO_2_ exhibiting a high discharge capacity of 790 mAh g^–1^, along with impressive cyclic stability for over 600 h. This work presents a promising strategy for the rational design of advanced ORR electrocatalysts, offering significant potential for ZABs and other energy-related applications.

The Pt/P-Mo_2_C catalyst was synthesized using heteropolyacid–pyrrole polymer (PMo_12_-PPy) nanospheres (NSs) as sacrificial templates. Chloroplatinic acid was employed as the Pt precursor, enabling one-step Pt loading onto phosphorus-doped Mo_2_C (P-Mo_2_C) via thermal reduction (see Experimental Section for details). Figures 1a, 1b, and S1 display representative SEM and TEM images of the PMo_12_-PPy NSs, showing a uniform spherical morphology with an average diameter of approximately 50 nm. After heat treatment, the resulting Pt/P-Mo_2_C retains the spherical morphology of the precursor and exhibits a well-defined core–shell structure, as shown in Figures 1c, 1d, and S2.

**Figure 1:**
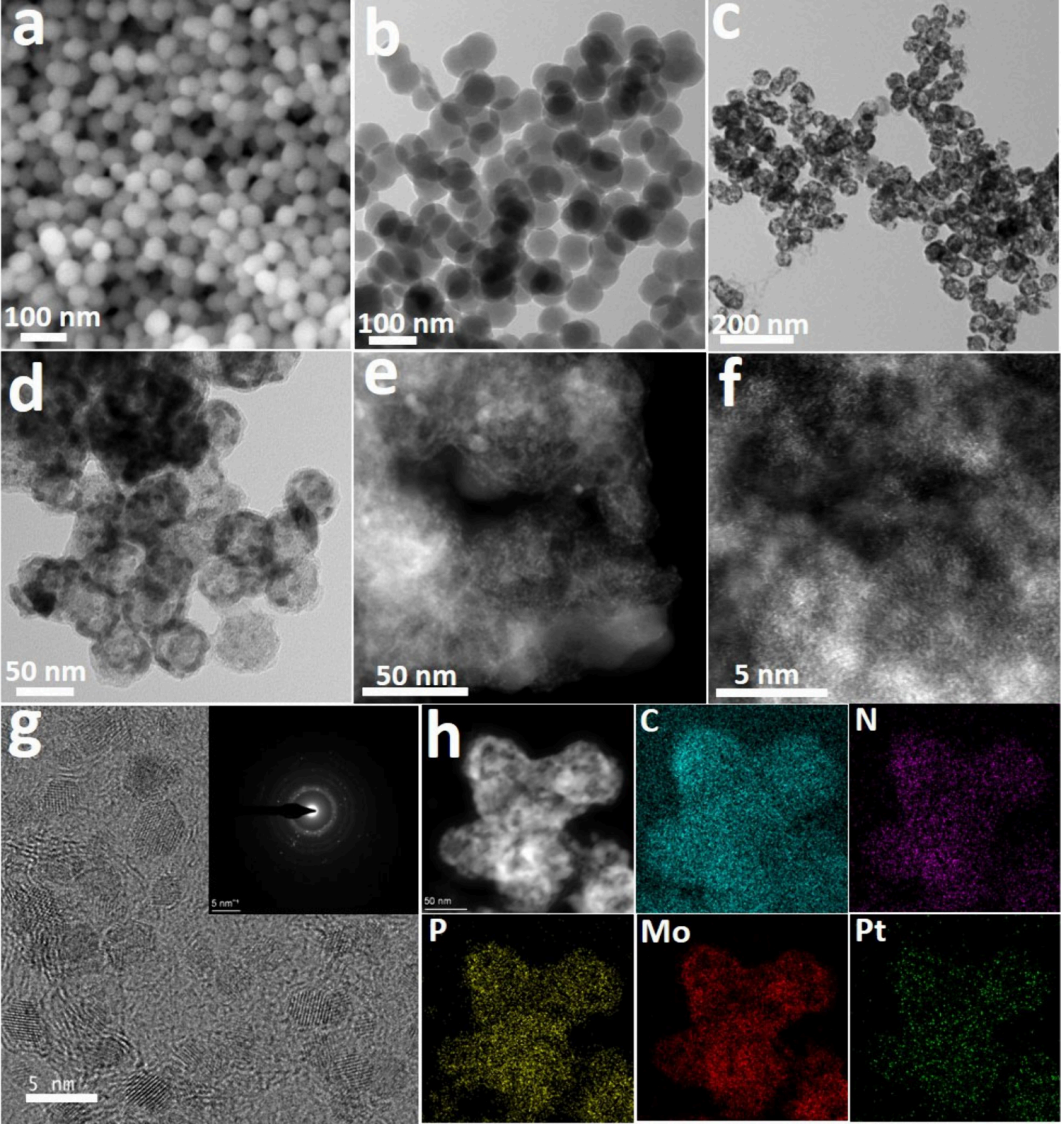
Structural and chemical characterization. a) SEM and b) TEM image of PMo_12_-PPy NSs. c,d) TEM images, e,f) AC-HAADF-STEM images (yellow circles highlight isolated bright spots corresponding to atomically dispersed Pt atoms), g) HRTEM images and corresponding FFT spectrum (inset), and h) HAADF-STEM and EELS chemical composition maps of Pt/P-Mo_2_C.

Atomic-resolution AC-HAADF-STEM images of Pt/P-Mo_2_C confirm the presence of atomically dispersed Pt single atoms (yellow circles highlight isolated bright spots corresponding to atomically dispersed Pt atoms) anchored on the surface of the P-Mo_2_C nanospheres (Figures 1e, 1f, and S3). HRTEM and the corresponding fast Fourier transform (FFT) pattern (Figure 1g) indicate that the Mo_2_C domains adopt a crystalline structure that is consistent with the cubic Mo_2_C phase. Additionally, HAADF-STEM imaging combined with EELS elemental mapping (Figure 1h) confirms the uniform distribution of C, N, P, Mo, and Pt throughout the nanostructure on the nanometer scale, evidencing the successful incorporation of all constituent elements.

As shown in Figures 2a and 2b, the XRD patterns of P-Mo_2_C and the Pt/P-Mo_2_C samples with varying Pt loadings confirm the formation of the β-Mo_2_C phase (PDF#11-0680), consistent with the HRTEM results.^29^ Notably, increasing Pt loading results in a gradual weakening of the Mo_2_C diffraction peaks, suggesting that the incorporation of Pt alters the crystallinity of the Mo_2_C lattice. This structural transformation may be attributed to the anchoring of Pt atoms, which introduces a lattice distortion. For comparison, in Pt/Mo_2_C synthesized without phosphorus doping, the Mo precursor converts to amorphous Mo_2_C (a-Mo_2_C), evidenced by a broad XRD peak characteristic of the amorphous phase.^25^

**Figure 2:**
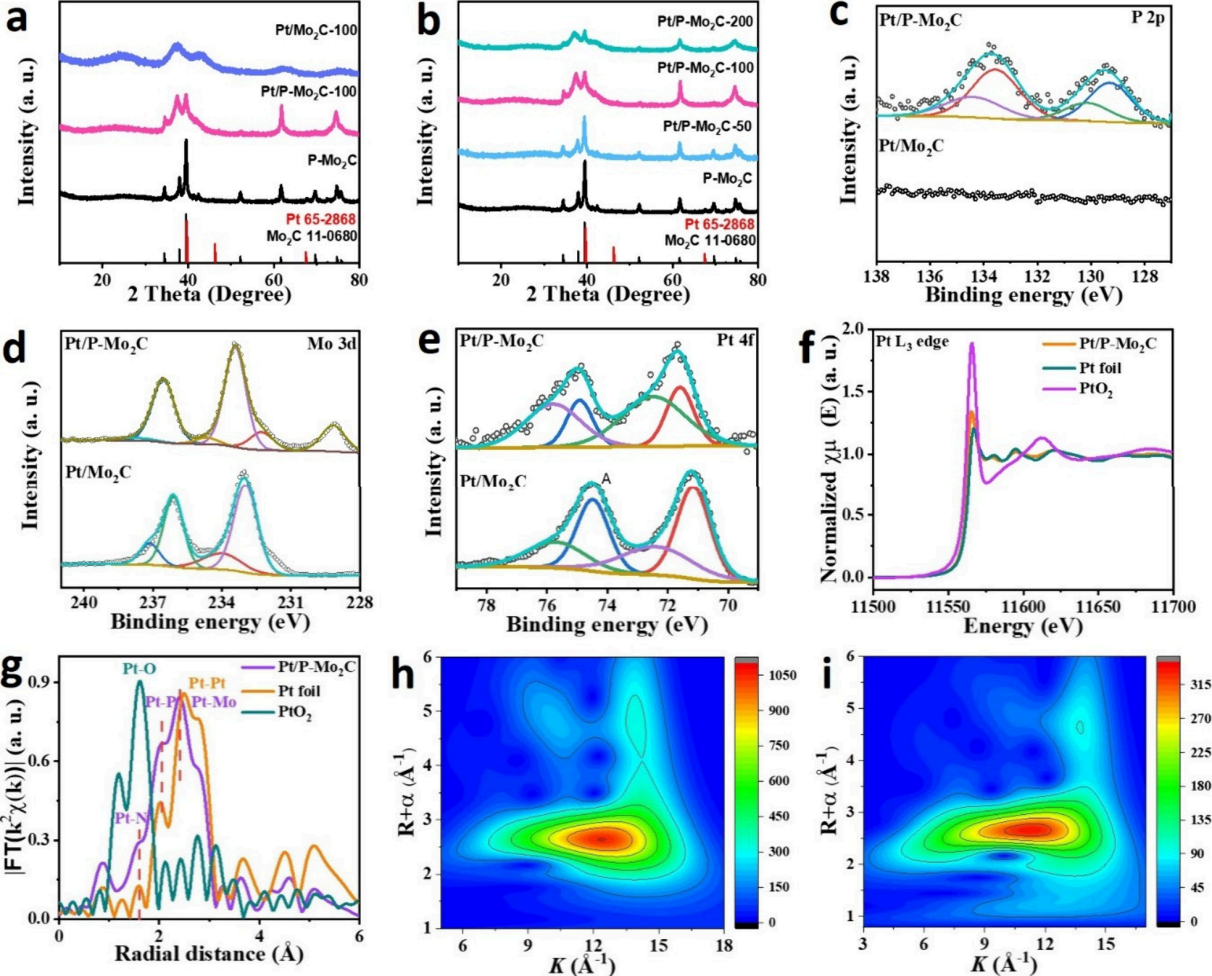
a,b) XRD patterns. c,d) High-resolution P 2p, Mo 3d, and Pt 4f XPS spectra of Pt/P-Mo_2_C and Pt/Mo_2_C. f) XANES Pt L_3_-edge spectra; g) FT curves of Pt/P-Mo_2_C, Pt foil, and PtO_2_; h,i) WT-EXAFS plots of Pt foil and Pt/P-Mo_2_C.

To investigate the chemical environment and local coordination of the catalyst, XPS and X-ray absorption fine structure (XAFS) measurements were performed. The high-resolution P 2p XPS spectrum (Figure 2c) reveals two pairs of peaks: the first at 129.5 and 130.1 eV corresponds to P 2p_3/2_ and P 2p_1/2_ from P in a metal phosphide environment,^30,31^ while the second set at 133.6 and 134.4 eV is attributed to oxidized phosphate species (P-O), formed due to surface oxidation upon air exposure, consistent with prior reports on metal phosphides.^32,33^

The high-resolution Mo 3d XPS spectrum (Figure 2d) of Pt/P-Mo_2_C shows new peaks at 229.0 and 232.1 eV, indicative of the Mo^3+^ species. These features are absent in Pt/Mo_2_C and suggest the formation of Mo–P bonds, introduced by phosphorus doping.^34–36^ In the Pt 4f region (Figure 2e), the spectrum can be deconvoluted into four peaks at 72.5 and 75.8 eV (Pt 4f_7/2_ and 4f_5/2_, respectively), which are slightly shifted from the typical metallic Pt^0^ signals (71.6 and 74.9 eV), indicating a partially oxidized Pt^δ+^ state, intermediate between Pt^0^ and Pt^2+^.^37–39^

Further insights into the Pt coordination environment were obtained via X-ray absorption near-edge structure (XANES) and extended X-ray absorption fine structure (EXAFS) spectroscopy. The Pt L_3_-edge XANES spectrum (Figure 2f) shows an absorption edge position consistent with a positively charged Pt species, in line with the XPS analysis. The Fourier-transformed EXAFS spectrum (Figure 2g) displays three dominant peaks located at 1.60 Å, 2.05 Å, and 2.40 Å, corresponding to Pt–N, Pt–P, and higher-shell Pt–Mo scattering paths, respectively.^25,40^ Notably, no Pt–Pt coordination peak is observed, unlike in the Pt foil, confirming the atomic dispersion of Pt.

To further verify this, wavelet transform (WT) analysis of the EXAFS oscillations was performed. While Pt foil exhibits a high-intensity maximum at 12.5 Å^–1^ associated with Pt–Pt coordination (Figure 2h), the WT contour map of Pt/P-Mo_2_C shows a dominant signal at 11.0 Å^–1^ (Figure 2i), consistent with Pt–Mo and Pt–P interactions and affirming the atomic dispersion of Pt.

Importantly, the atomic embedding of Pt in the P-doped Mo_2_C matrix is predicted to enhance the ORR kinetics, particularly in alkaline environments. This is attributed to charge redistribution at the Mo–Pt–P sites, where electron donation from Pt to Mo_2_C leaves positively charged Pt centers, acting as Lewis acid sites. These sites facilitate hydroxyl adsorption and promote the Volmer step, thereby accelerating the alkaline ORR process.

The ORR performance of Pt/P-Mo_2_C, Pt/Mo_2_C, and P-Mo_2_C with varying Pt contents, and commercial Pt/C was evaluated using a conventional three-electrode system in 0.1 M KOH alkaline media. A Hg/HgO (1 M KOH) calibrated with respect to the reversible hydrogen electrode (RHE) (Figure S4) and platinum wire were employed as reference and counter electrodes, respectively. As shown in Figures 3a and 3b, Pt/P-Mo_2_C-100 exhibits exceptional catalytic activity, with a half-wave potential (*E*_1/2_) of 0.91 V. These values are significantly higher than those of Pt/Mo_2_C (*E*_1/2_ = 0.86 V) and P-Mo_2_C (*E*_1/2_ = 0.78 V), underscoring the synergistic effect of Pt single atoms and phosphorus doping. Remarkably, Pt/P-Mo_2_C-100 also surpasses the commercial Pt/C benchmark (*E*_1/2_ = 0.89 V) and outperforms many other reported ORR electrocatalysts under similar conditions (Table S1).

**Figure 3:**
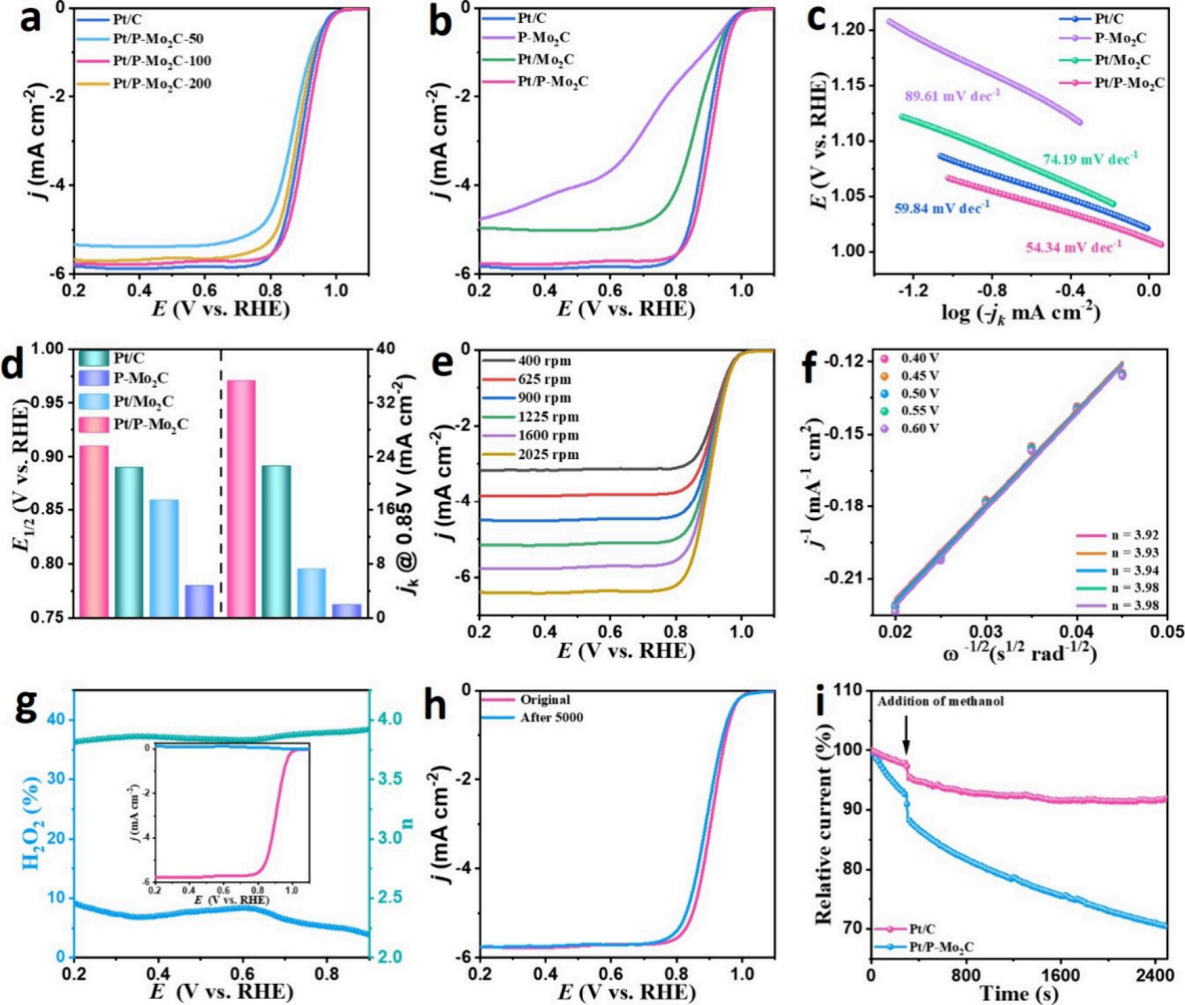
a, b) LSV curves, c) corresponding Tafel plots, and d) *E*_half_ and *j*_k_ (at 0.85 V vs RHE) of Pt/P-Mo_2_C, Pt/Mo_2_C, P-Mo_2_C, and Pt/C catalysts for ORR in O_2_-saturated 0.1 M KOH at 1600 rpm; e) LSV curves recorded at different rotating speeds; f) corresponding K–L plots; g) electron transfer number *n* and H_2_O_2_ yield (inset: rotating ring disk electrode LSV curves at 1600 rpm); h) LSV curves before and after 5000 CV cycles of Pt/P-Mo_2_C. (i) Chronoamperometric measurements and methanol tolerance evaluation of Pt/P-Mo_2_C and commercial Pt/C at 0.7 V vs RHE.

Tafel slope analysis (Figure 3c) was performed to investigate the ORR kinetics. Pt/P-Mo_2_C-100 displays the lowest Tafel slope of 54.34 mV dec^–1^, compared to Pt/C (59.84 mV dec^–1^), Pt/Mo_2_C (74.19 mV dec^–1^), and P-Mo_2_C (89.61 mV dec^–1^), indicating superior reaction kinetics and faster electron transfer on Pt/P-Mo_2_C-100.^41,42^

The kinetic current density (*j*_k_), derived from the polarization curves at 0.85 V (Figure 3d), further confirms the enhanced performance of Pt/P-Mo_2_C-100, which achieves a *j*_k_ of 35.22 mA cm^–2^. This is notably higher than those of Pt/C (22.66 mA cm^–2^), Pt/Mo_2_C (7.28 mA cm^–2^), and P-Mo_2_C (2.01 mA cm^–2^), validating the effective utilization of active sites facilitated by Pt single atom dispersion. Phosphorus incorporation modulates the electronic structure of Pt, weakening the binding strength of *OH and *O intermediates, thereby accelerating ORR kinetics.

To elucidate the reaction mechanism, linear sweep voltammetry (LSV) was recorded at various rotation speeds of the rotating disk electrode (RDE) (Figure 3e). The resulting Koutecky–Levich (K–L) plots (Figure 3f) demonstrate linearity at multiple potentials (0.40–0.60 V), indicating first-order kinetics with respect to O_2_. The electron transfer number (*n*) calculated from the K–L plots approaches 4, consistent with a four-electron ORR pathway (O_2_ + 2H_2_O + 4e^–^ → 4OH^–^).^43–45^ Rotating ring-disk electrode (RRDE) measurements further validate this, with an *n* value of 3.81 and the hydrogen peroxide yield remaining below 9.17% across the potential range (Figure 3g), demonstrating high selectivity for the preferred four-electron reduction route, which is also consistent with commercial Pt/C (yielding *n* = 3.97 and H_2_O_2_ yield = 10.3% in Figure S5).

Catalyst durability was evaluated through an accelerated degradation test (ADT). As shown in Figure 3h, Pt/P-Mo_2_C experiences only a 12 mV loss in *E*_1/2_ after 5000 cycles, indicating outstanding electrochemical stability. In contrast, Pt/C exhibited a more significant performance decay. Additionally, the methanol crossover resistance, a crucial parameter for fuel cell applications, was assessed. Upon methanol injection (Figure 3i), the current response of Pt/P-Mo_2_C remained stable, while Pt/C displayed a sharp decline, confirming the superior ORR selectivity and methanol tolerance of Pt/P-Mo_2_C.

Collectively, these results demonstrate that the engineered Pt/P-Mo_2_C catalyst exhibits exceptional ORR activity, superior reaction kinetics, long-term durability, and strong tolerance to methanol poisoning, outperforming commercial Pt/C and other reference materials. These advantages are attributed to the synergistic effects of atomically dispersed Pt and phosphorus-modified Mo_2_C, which together enhance active site accessibility, optimize intermediate binding energies, and accelerate reaction kinetics.

To uncover the origin of the outstanding ORR activity of Pt/P-Mo_2_C, DFT calculations were performed. Guided by experimental results from XRD, TEM, HAADF-STEM, and XAFS, an optimized atomic model of the Pt/P-Mo_2_C heterostructure was constructed (Figure S6b). For comparison, a model of Pt/Mo_2_C without phosphorus doping was also developed (Figure S6a).

The calculated density of states (DOS) for both systems (Figure 4b) reveals a significantly higher DOS at the Fermi level for Pt/P-Mo_2_C compared to Pt/Mo_2_C. This indicates enhanced electron delocalization at the interface, which facilitates improved charge transfer and promotes electrocatalytic activity.^46,47^ The elevated DOS near the Fermi level also suggests better electrical conductivity and a reduced activation barrier for electron donation to adsorbed O_2_ molecules, thereby accelerating the ORR process.

**Figure 4:**
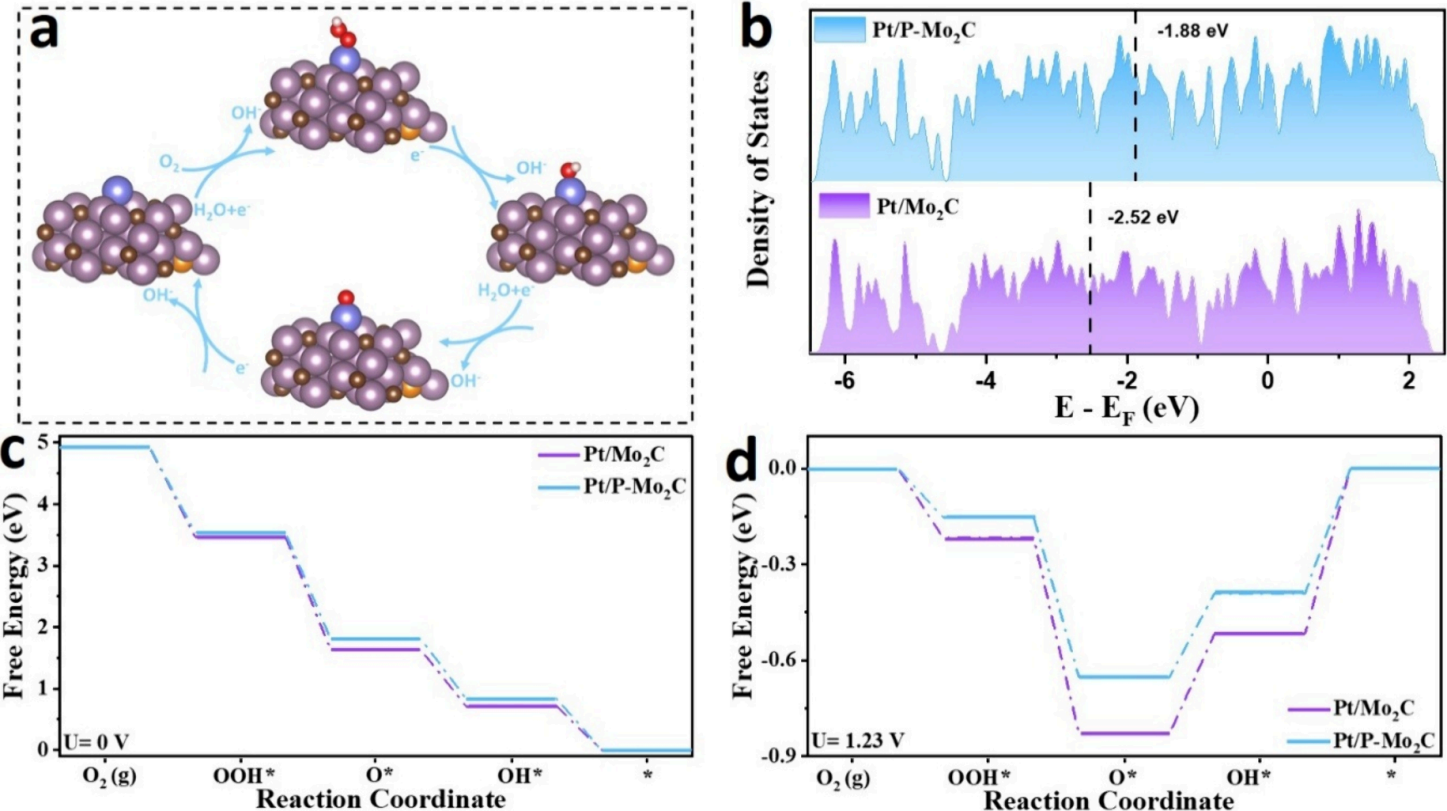
a) Evolution of the local structural configurations illustrating the ORR process of Pt/P-Mo_2_C. b) d-band centers of DOS and c,d) Gibbs free energy diagrams of ORR intermediates for Pt/P-Mo_2_C and Pt/Mo_2_C.

According to d-band center theory,^48^ moderate binding strength of oxygen intermediates is critical for optimal catalytic performance. In this context, Pt/P-Mo_2_C exhibits a d-band center at −1.88 eV, higher than that of Pt/Mo_2_C (−2.52 eV), indicating a more favorable interaction with ORR intermediates and a likely enhancement in the overall activity.

Charge density difference analysis further suggests significant charge redistribution at the Pt/P-Mo_2_C interface, which effectively tunes the adsorption energies of oxygen-containing intermediates (*OOH, *O, and *OH) and helps to lower the reaction overpotentials. Figure 4a illustrates the atomic configurations of these intermediates along the four-electron ORR pathway.

The ORR free energy diagrams for both catalysts at different applied potentials are shown in Figures 4c and 4d. At *U* = 0 V, both systems exhibit downhill free energy profiles, confirming that the overall ORR processes are thermodynamically favorable. However, at the practical operating potential of *U* = 1.23 V, the rate-determining step (RDS) on Pt/P-Mo_2_C exhibits a lower energy barrier of 0.61 eV, compared to a higher barrier on Pt/Mo_2_C. This demonstrates that the phosphorus-doped interface of Pt/P-Mo_2_C not only optimizes the adsorption of reaction intermediates but also significantly reduces the energy barrier associated with the RDS, leading to enhanced catalytic performance.

In summary, the DFT calculations validate that the Pt/P-Mo_2_C heterostructure enables effective electronic reconfiguration, facilitates favorable oxygen intermediate adsorption, and lowers kinetic barriers along the ORR pathway, all of which contribute to its superior electrocatalytic activity.

Based on the outstanding ORR activity of the Pt/P-Mo_2_C catalyst in alkaline media, ZABs were assembled using a homemade cell configuration. The Pt/P-Mo_2_C catalyst, paired with commercial RuO_2_, served as the air cathode, while a benchmark device using commercial Pt/C and RuO_2_ was assembled for comparison. Figure 5a illustrates a schematic of the ZAB device.

**Figure 5:**
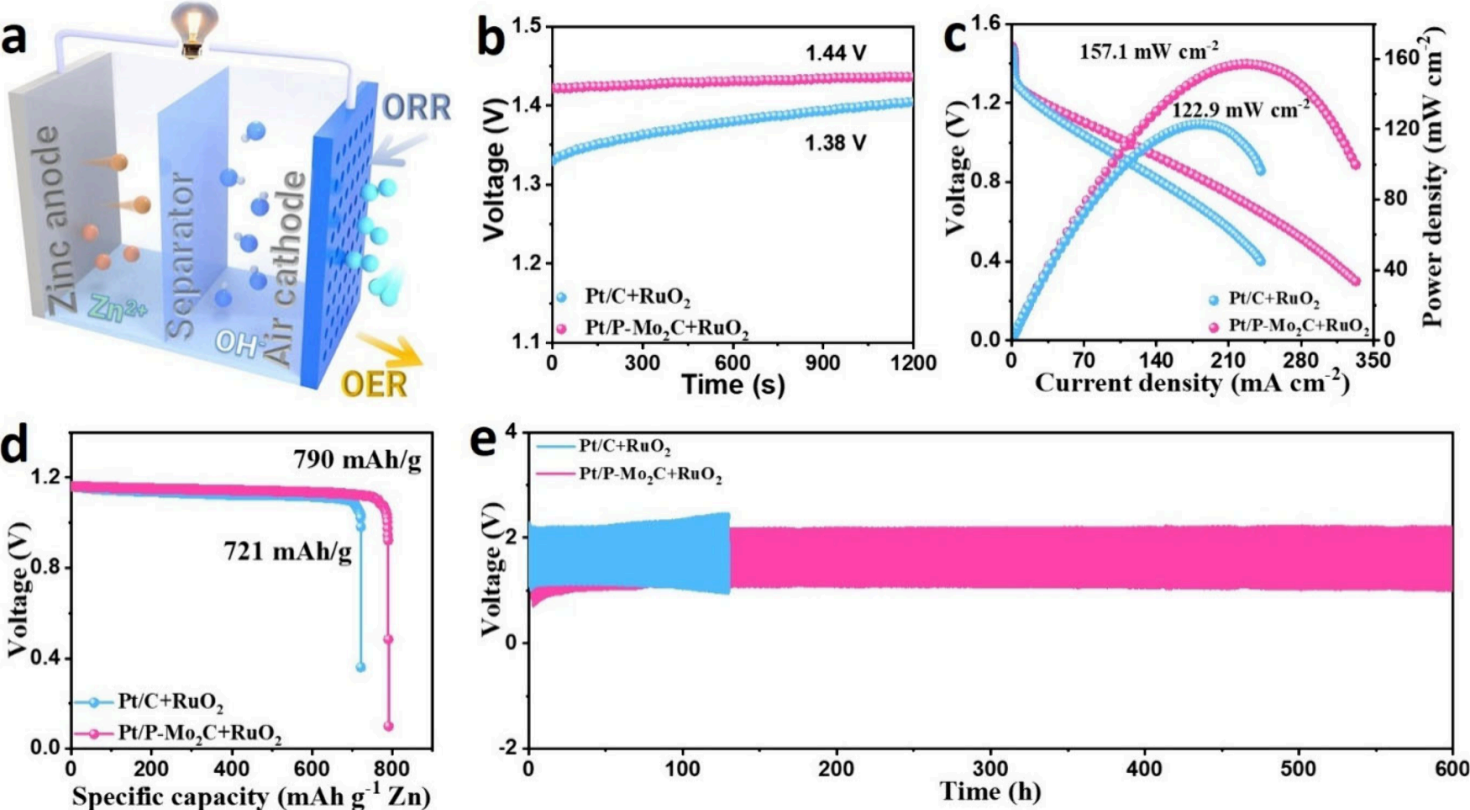
a) Schematic diagram of aqueous ZAB, b) open-circuit voltage, c) discharge polarization curves and corresponding peak powder density, d) corresponding specific capacities, and e) long-term charge–discharge cycling stabilities of Pt/P-Mo_2_C + RuO_2_ and Pt/C + RuO_2_.

As shown in Figure 5b, the ZAB equipped with Pt/P-Mo_2_C and RuO_2_ exhibits a higher open-circuit voltage (OCV) of 1.44 V, compared to 1.38 V for the Pt/C-based cell, indicating a better electrochemical potential. Discharge polarization and power density curves (Figure 5c) reveal that the Pt/P-Mo_2_C-based cell delivers a superior peak power density of 157.1 mW cm^–2^ at a current density of 229 mA cm^–2^, significantly outperforming the Pt/C-based device, which reaches 122.9 mW cm^–2^ at 179 mA cm^–2^. This enhanced performance underscores the catalytic superiority of Pt/P-Mo_2_C under practical operating conditions.

The specific capacity, normalized to zinc consumption during discharge at 10 mA cm^–2^ (Figure 5d), reaches 790 mAh g^–1^ Zn for the Pt/P-Mo_2_C-based ZAB, surpassing that of the Pt/C-based system (721 mAh g^–1^ Zn), further confirming its efficient utilization of active materials. Most notably, the Pt/P-Mo_2_C-based ZAB demonstrates excellent cycling stability, maintaining a stable charge–discharge profile over 1200 cycles (600 h) at 10 mA cm^–2^ with minimal voltage decay (Figure 5e). In contrast, the Pt/C-based ZAB shows significant performance degradation, with a widened charge–discharge voltage gap after only 100 h of operation, highlighting the superior long-term durability of the Pt/P-Mo_2_C catalyst in rechargeable ZABs.

In this work, we successfully designed and synthesized a single-atom platinum electrocatalyst supported on phosphorus-doped molybdenum carbide (Pt/P-Mo_2_C) through a confined polymerization and thermal reduction strategy. The resulting heterostructure features atomically dispersed Pt sites embedded within a conductive P-Mo_2_C matrix, as confirmed by advanced microscopy and spectroscopic analyses. The introduction of nonmetallic phosphorus plays a critical role in modifying the electronic structure of Mo_2_C, enabling strong interfacial electronic coupling with Pt atoms and stabilizing the single-atom configuration. DFT calculations reveal significant charge redistribution at the Pt/P-Mo_2_C interface, which modulates the d-band center of Pt, lowers energy barriers for oxygen intermediate adsorption/desorption (*OH, *OOH, and *O), and enhances the overall ORR kinetics. As a result, the Pt/P-Mo_2_C catalyst demonstrates remarkable electrocatalytic performance in alkaline media, with a high half-wave potential (*E*_1/2_ = 0.91 V), superior kinetic current density (*j*_k_ = 45.27 mA cm^–2^ at 0.85 V), and excellent durability over 5000 cycles with minimal performance decay. Furthermore, the catalyst exhibits strong methanol tolerance and four-electron selectivity, as evidenced by low peroxide yields and an electron transfer number close to 4. When implemented in a rechargeable ZAB, Pt/P-Mo_2_C achieves an open-circuit voltage of 1.44 V, a high peak power density of 157.1 mW cm^–2^, and a large specific capacity of 790 mAh g^–1^ of Zn. Most notably, the assembled ZAB maintains excellent cycling stability over 600 h and 1200 cycles, significantly outperforming a reference device based on commercial Pt/C and RuO_2_. Overall, this study highlights the critical role of phosphorus doping in engineering electronic interfaces and stabilizing single-atom active sites. The Pt/P-Mo_2_C heterostructure offers a promising pathway for the development of cost-efficient, durable, and high-performance electrocatalysts for both ORR and energy storage applications, particularly in metal–air batteries and fuel cells.

## Supplementary Material



## Data Availability

All the data that support the findings of this study are available from the corresponding author upon request.

## Notes

Experimental Section, characterization, electrochemical measurements, zinc–air battery measurements, theoretical modeling and calculations, SEM, TEM, AC-HAADF-STEM, characterization results of the corresponding electrocatalysts, the data of electrochemical tests, local structural configurations of corresponding electrocatalysts, and a table summarizing the ORR activity for some reported electrocatalysts (PDF)
