# Supplementary material for: Enabling Efficient Oxygen Reduction Reaction with Pt Single Atoms on Carbide: A Phosphorus-Doped Mo2C Interface Strategy
Source: Nano Lett. 2025 Oct 29;25(45):16187–95. doi: 10.1021/acs.nanolett.5c04201 (PMC12616767; doi:10.1021/acs.nanolett.5c04201)
Supplement: Supplementary file 1 [file nl5c04201_si_001.pdf]

Support Information

Enabling Efficient Oxygen Reduction Reaction with Pt Single Atoms on Carbide: A Phosphorus-Doped Mo_2C Interface Strategy

Changwei Shi,[†, ‡] Xingmao Jiang,[†, ‡] Xueqiang Qi,[§, ⊥] Congcong Xing,[ǁ] Xiaolei Fan,[ǁ, ¢, ○] Zhuo Chen,[ȡ] Xiang Wang,*[, †, ‡, §] Andreu Cabot*[, §, ¶]

[†] School of Chemical Engineering and Pharmacy, Hubei Key Laboratory of Novel Reactor and Green Chemical Technology, Wuhan Institute of Technology, Wuhan, 430205, China

[‡] State Key Laboratory of Green and Efficient Development of Phosphorus Resources, Wuhan Institute of Technology, Wuhan, 430205, China

[§] Catalonia Institute for Energy Research (IREC), Sant Adrià de Besòs, 08930 Barcelona, Spain

[⊥] School of Chemistry and Chemical Engineering, Chongqing University, Chongqing 400044, China

[ǁ] Wenzhou Key Laboratory of Novel Optoelectronic and Nano Materials, Institute of Wenzhou, Zhejiang University, Wenzhou 325006, China

[¢] Department of Materials Science and Engineering, Zhejiang University, Hangzhou 310027, China

[○] Department of Chemical Engineering, School of Engineering, The University of Manchester, Oxford Road, Manchester M13 9PL, United Kingdom

[ȡ] State Key Laboratory of Advanced Technology for Materials Synthesis and Processing Wuhan University of Technology, Wuhan 430070, P. R. China

[¶] ICREA Pg. Lluis Companys, 08010 Barcelona, Catalonia, Spain

* E-mails: X.Wang: wangxiang@wit.edu.cn; A. Cabot: acabot@irec.cat

Experimental Section

Chemicals

Chloroplatinic acid hydrate ($H_2PtCl_6 \cdot xH_2O$, Pt 37-40%), phosphomolybdic acid ($H_3PMo_{12}O_{40} \cdot nH_2O$, PMo_{12}, ≥99%), ammonium heptamolybdate tetrahydrate ($(NH_4)_6Mo_7O_{24} \cdot 4H_2O$), potassium hydroxide (KOH, 85%), zinc acetate dihydrate ($Zn(CH_3COO)_2 \cdot 2H_2O$, 98%), hydrochloric acid (HCl, 37%) Pyrrole (C_4H_5N, ≥98%) and Nafion solution (5 wt% in a mixture of low aliphatic alcohols and water) were obtained from Sigma-Aldrich. The reference Pt catalyst, 20% Pt on activated carbon powder, was purchased from Alfa Aesar. Ethanol was of analytical grade and obtained from various sources. MilliQ water was obtained from a Purelab flex from Elga. All chemicals were used as received, without further purification.

Synthesis of PMo_{12}-PPy NSs

420 µL pyrrole solution was added in 25 mL absolute alcohol, then the formed solution was added dropwise to phosphomolybdic acid aqueous solution (2.19 g in 100 mL of deionized water) with continuous stirring at room temperature. After reacting for 12 h, the precipitate was collected by filtering and vacuum dried to obtain heteropolyacid pyrrole polymer nanospheres, named PMo_{12}-PPy NSs.

Synthesis of P-Mo_2C, Pt@P-Mo_2C and Pt@Mo_2C

200 mg PMo_{12}-PPy NSs were dispersed in 10 mL deionized water by ultrasonication, and then 50, 100, or 200 µL of 0.1 g ml^{-1} chloroplatinic acid solution was added to the above solution. The precipitate was collected by centrifugation after continuous ultrasonic treatment for 20 min and vacuum dried. Finally, the obtained powder was calcined at 900 °C for 2 h under Ar atmosphere with a ramp rate of 5 °C min^{-1} to get the final product platinum nanoclusters supported on phosphorus-doped molybdenum carbide, named Pt/P-Mo_2C-50, Pt/P-Mo_2C-100, Pt/P-Mo_2C-200, respectively. Phosphorus-doped molybdenum carbide (P-Mo_2C) was obtained by directly calcining PMo_{12}-PPy NSs without further processing.

Platinum nanoclusters supported on molybdenum carbide (Pt/Mo_2C) were prepared by following the same procedure for the preparation of Pt/P-Mo_2C except for adding 4.96 g of ammonium heptamolybdate tetrahydrate ($(NH_4)_6Mo_7O_{24} \cdot 4H_2O$) into 80 mL of deionized water containing 6.6 mL of Py, followed by adjusting the pH to 1.5 with 1 M HCl.

Structural characterization

Powder X-ray diffraction (XRD) was performed on a Bruker AXS D8 Advance X-ray diffractometer with Cu-Kα radiation (λ= 1.5406 Å). Scanning electron microscopy (SEM) analysis was conducted with a Zeiss Auriga microscope equipped with an energy dispersive spectroscope analyses (EDS) detector operating at 20 kV. Transmission electron microscopy (TEM), High-resolution TEM (HRTEM), Annular dark-field scanning transmission electron microscope (HAADF-STEM) and electron energy loss spectroscopy (EELS) analysis were obtained using a field emission gun FEI™ Tecnai F20 microscope with a Gatav Quantum filter at 200 kV. X-ray photoelectron spectroscopy (XPS) measurements were conducted on a SPECS system equipped with an Al anode XR50 source operating at 150 W and a Phoibos 150 MCD-9 detector. The spectral data of the X-ray absorption fine structure (XAFS) were measured at the 1W1B station of Beijing Synchrotron Radiation Facility (BSRF).

Reversible hydrogen electrode (RHE) calibration : We used Hg/HgO (1.0 M KOH) electrode as the reference electrode in all measurements. It was calibrated with respect to reversible hydrogen electrode (RHE). The calibration was performed in the high purity hydrogen saturated electrolyte (0.1 M KOH) with a Pt wire as the working electrode. CVs were run at a scan rate of 1 mV s^{-1}, and the average of the two potentials at which the current crossed zero was taken to be the thermodynamic potential for the hydrogen electrode reactions.

Electrochemical Measurements

The electrochemical performance was evaluated in 0.1 M KOH on a rotating ring-disk electrode by CHI760E electrochemical workstation. A Hg/HgO (1 M KOH) and platinum wire were employed as reference and counter electrodes, respectively. The catalyst ink was produced by ultrasonically dispersing 4 mg of the catalyst powder in 750 µL isopropanol, 230 µL deionized water and 20 µL of 5 wt% Nafion solution. The working electrode was prepared by drop-casting 5 µL of the catalyst ink on electrode and letting it dry naturally. Before tests, O_2 or Ar was flowed through the electrolyte for about 30 min to achieve the saturated solution. Cyclic voltammetry (CV) curves were measured in O_2-saturated or Ar-saturated solution with a scan rate of 5 mV/s. Linear sweep voltammetry (LSV) tests were conducted in O_2-saturated electrolyte at different rotation rates with a sweep rate of 5 mV/s.

Rotating disk electrode (RDE) measurement.

The working electrode (RDE with a diameter 5 mm) was scanned cathodically at a rate of 5 mVs^{-1} with varying rotating speed from 400 rpm to 2025 rpm. Koutecky–Levich plots (J^{-1} vs. $\omega^{-1/2}$) in the insets of Figure 2 of the main text were analyzed at various electrode potentials. The slopes of their best linear fit lines were used to calculate the number of electrons transferred (n) on the basis of the Koutecky-Levich equation1:

$$\frac{1}{J} = \frac{1}{J_L} + \frac{1}{J_K} = \frac{1}{B\omega^{1/2}} + \frac{1}{J_K}$$

$$\mathrm{B} = 0.62nFC_0(D_0)^{2/3}v^{1/6}$$

$$J_K = nFkC_0$$

where J is the measured current density, JK and JL are the kinetic- and diffusionlimiting current densities, ω is the angular velocity, n is transferred electron number, F is the Faraday constant, Co is the bulk concentration of O_2, v is the kinematic viscosity of the electrolyte, and k is the electron-transfer rate constant. For the kinetic current was calculated from the mass-transport correction of RDE by:

$$J_K = \frac{J \times J_L}{J_L - J}$$

Rotating ring-disk electrode (RRDE) measurement. For the RRDE measurements, catalyst inks and electrodes were prepared by the same method as RDE's (RRDE with a diameter of 4 mm for disk electrode). The ink was dried slowly in air and the drying condition was adjusted by trial and error until a uniform catalyst distribution across the electrode surface was obtained. The disk electrode was scanned cathodically at a rate of 5 mVs^{-1} and the ring potential was constant at 1.5 V vs RHE. The H_2O_2% and the electron transfer number (n) were determined by the followed equations:

$$\mathrm{n} = 4 \times \frac{I_d}{I_d + I_r/N}$$

$$\mathrm{H_2O_2\%} = 200 \times \frac{I_r/N}{I_d + I_r/N}$$

where I_d is disk current, I_r is ring current and N is current collection efficiency (N) of the Pt ring. N is the collection efficiency by determining to be 0.37 from the reduction of $K_3Fe[CN]_6$.

Zinc-air batteries (ZAB) assembly and test: the catalytic ink was sprayed on the hydrophobic carbon paper uniformly with a mass loading of 0.5 mg/cm^2. This carbon paper was used as the air cathode while a polished Zn foil was used as the anode. The two electrodes were assembled into a ZAB, while a 6 M KOH and 0.2 M zinc acetate aqueous solution was used as the electrolyte. Charge–discharge polarization curves and open circuit voltage measurements were determined by CHI760E electrochemical workstation. The galvanostatic test was performed using a Neware BTS4008 battery test system. The specific capacities were determined using the galvanostatic discharge profiles standardized to the consumed mass of Zn.

Theoretical modelling and calculations

The DFT calculations were conducted using the Vienna Ab initio Simulation Package (VASP) code. The ion core potentials were modelled following the projector augmented wave (PAW) approach to describe the electron−core interaction. For electron exchange−correlation, the Perdew−Burke−Ernzerhof (PBE) functional within the generalized-gradient approximation (GGA) range was applied. Calculations were performed with the cutoff plane-wave kinetic energy of 400 eV, and k-points were sampled using the 3 × 3 × 1 Monkhorst–Pack mesh. The original cell model of Pt/P-Mo2C was constructed with fully optimized atomic positions, cell volume and cell shape under conditions consistent with the experimental results. The vacuum spacing is 15 Å in the direction perpendicular to the catalyst plane. The maximum stress on each atom is within 0.02 eV $Å^{-1}$ under the quasi-Newtonian algorithm, and the equilibrium lattice constants are optimized

To better describe the dispersion interaction within water adsorption systems, vdW correction was considered by adopting Grimme's D3(BJ) scheme. A vacuum layer of 15 Å was employed to prevent interaction between slabs. The lower-half two layers of the slab were kept frozen and the upper-half two layers were allowed to relax. The adsorption energy E_{ads} on the different substrates was calculated according to the equation:

$$\Delta E = E_{(X/slab)} - E_{(slab)} - E_{(X)}$$

The free energies (ΔG) are calculated using the following equation:

$$\Delta G = \Delta E + \Delta ZPE - T\Delta S + \Delta G_U$$

Where, ΔE, ΔZPE, $\Delta U(0 \rightarrow T)$, $T\Delta S$, andΔG_U are the adsorption energy of adsorbed species on the given unit cell, the differences corresponding to the zero-point energy, the difference of the correction to

the thermal energy, the differences corresponding to the entropy (at 298.15 K), and the differences corresponding to the pH, respectively.

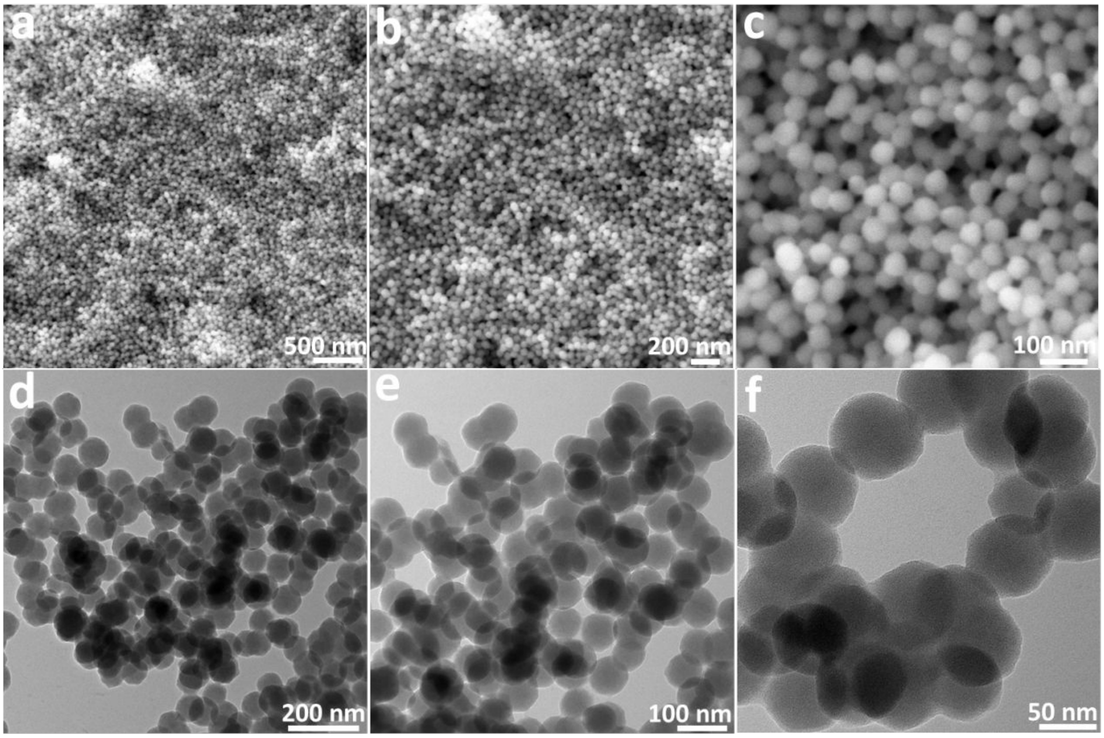

Figure S1. a-c) SEM and d-f) TEM images of PMo12-PPy.

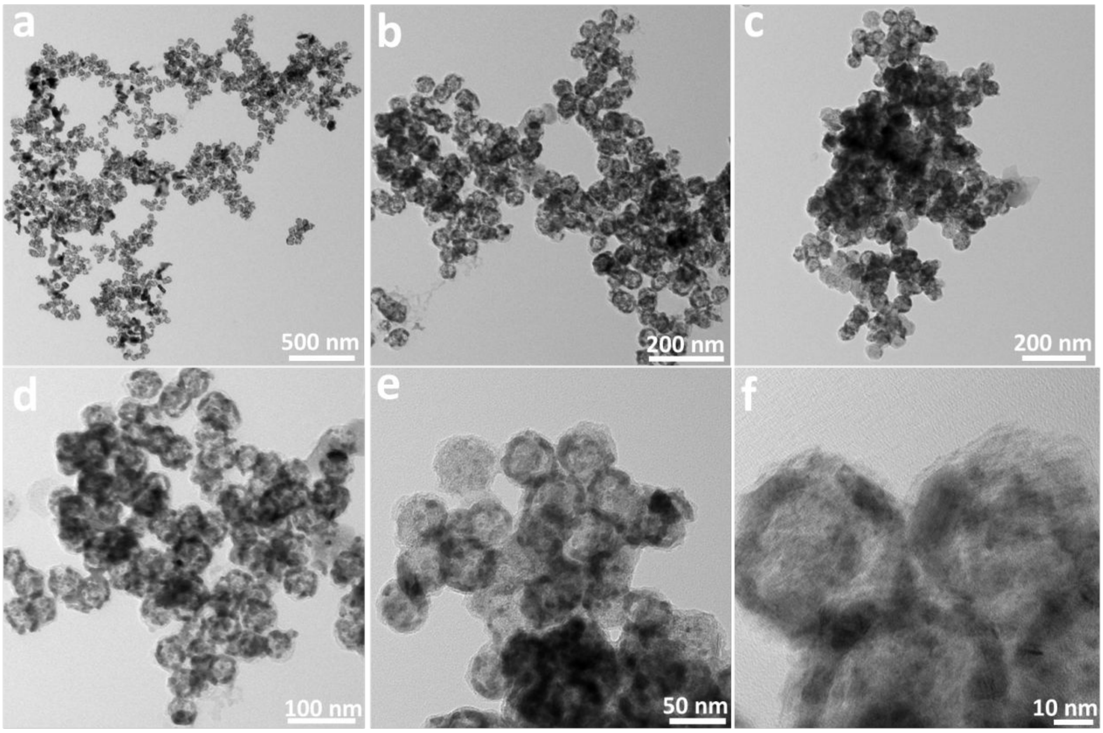

Figure S2. a-f) TEM images of Pt/P-Mo_2C.

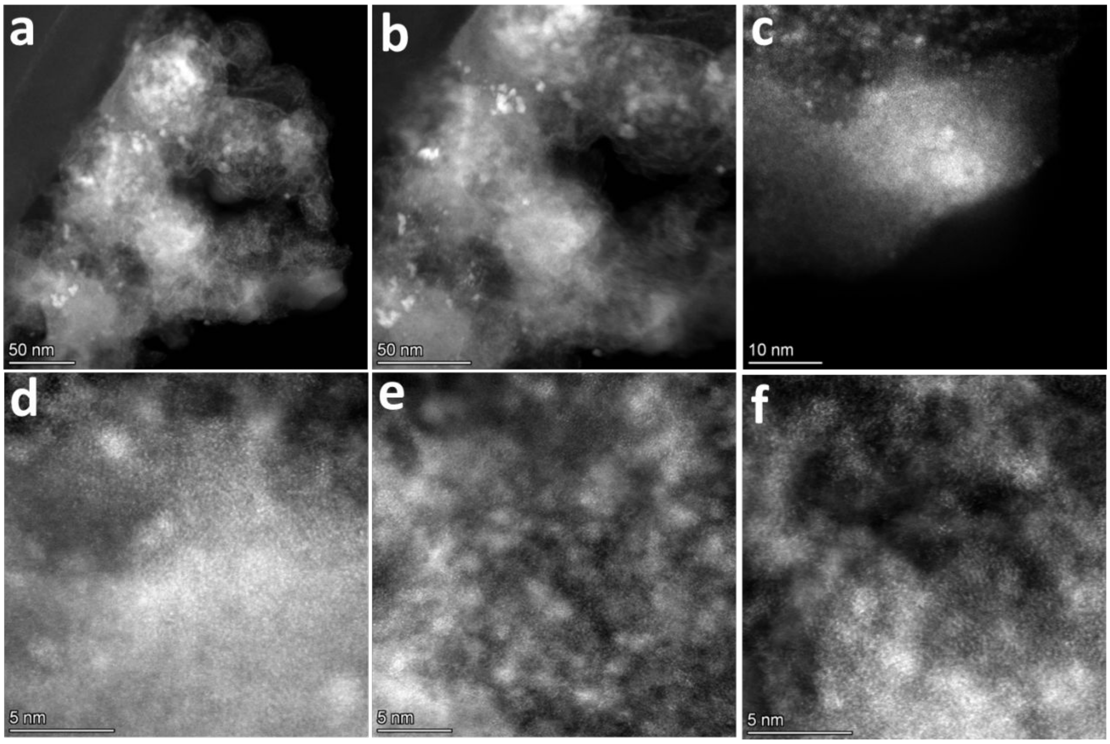

Figure S3. a-f) AC-HAADF-STEM images of Pt/P-Mo_2C.

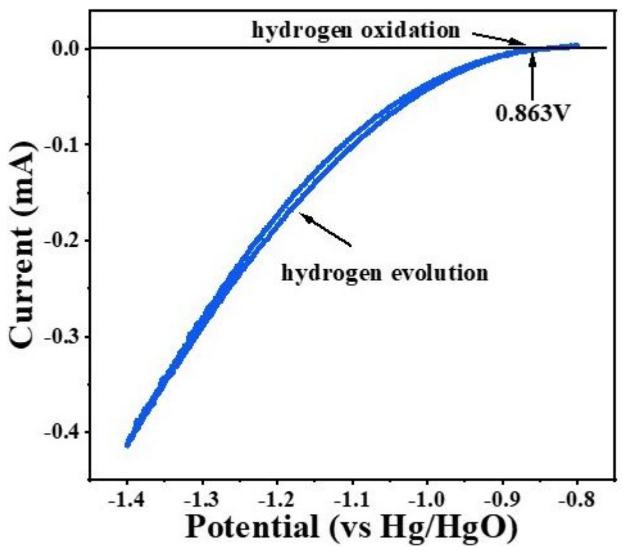

In 0.1 M KOH, E (RHE) = E (Hg/HgO) + 0.863 V.

Figure S4 The CV curve for reference electrode calibration curve in 0.1 M KOH electrolyte

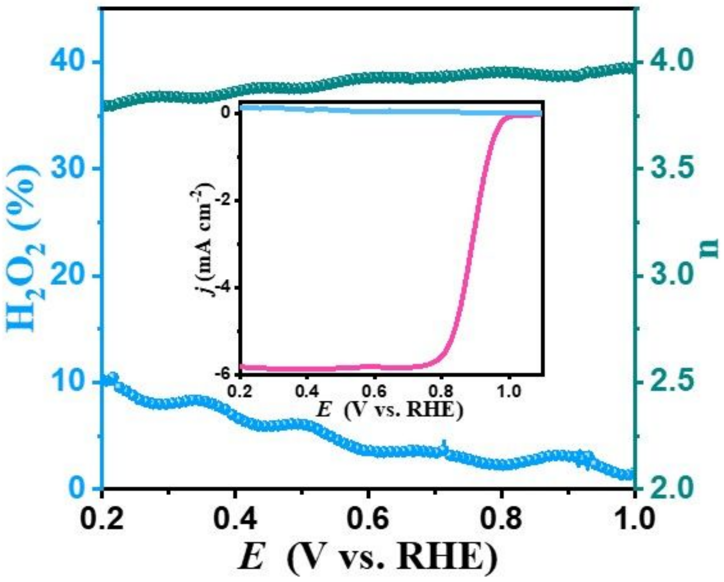

Figure S5 The electron transfer number n and H_2O_2 yield (inset: rotating ring disk electrode LSV curves at 1600 rpm) for commercial 20 wt.% Pt/C。

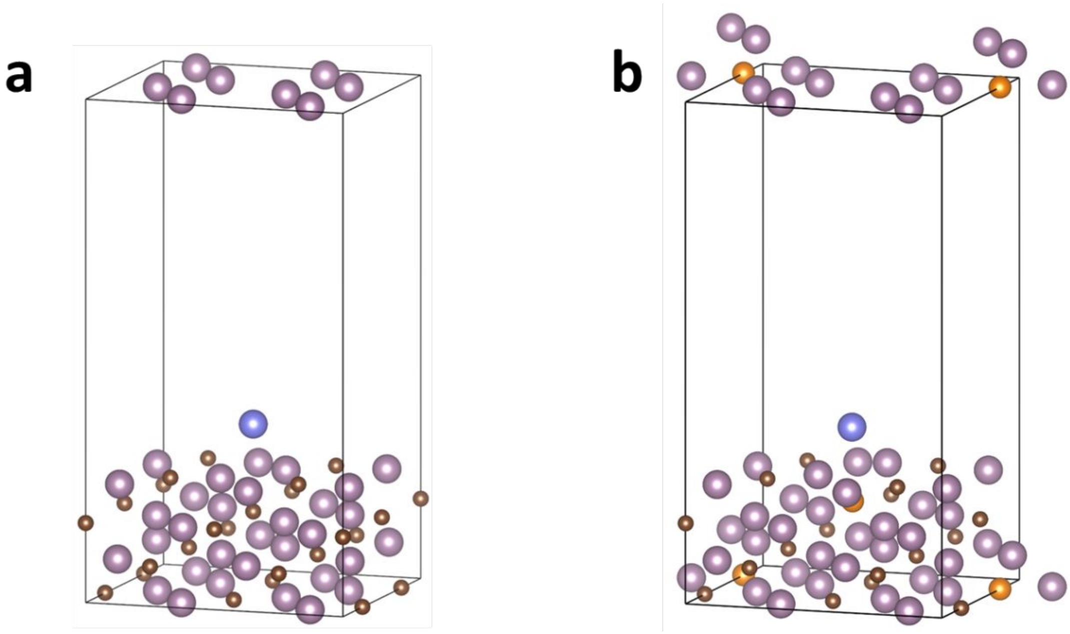

Figure S6. a-b) The local structural configurations of Pt/Mo_2C and Pt/P-Mo_2C.

Table S1. Summary of the ORR activity for some reported electrocatalyst.

Electrocatalyst	Electrolyte	$E_{1/2}$ (V vs. RHE)	Reference
**Pt/P-Mo_2C**	**0.1 M KOH**	**0.91**	**This work**
FeNC-VN	0.1 M KOH	0.90	J. Am. Chem. Soc. 2024, 146, 4803-4813 10949
FeN_4-Fe_{NCP}@MCF	0.1 M KOH	0.89	Adv. Funct. Mater. 2024, 2315150.
2H-$Pd_{67}Cu_{33}$ NPs	0.1 M KOH	0.90	J. Am. Chem. Soc. 2021, 143, 41, 17292-17299
T-Fe SAC	0.1 M KOH	0.91	Angew. Chem. Int. Ed. 2024, 63, 202319370
H-3DOM-Co/ONC	0.1 M KOH	0.84	Adv. Mater. 2023, 35, 2301894
P-O/FeNC-SAC	0.1 M KOH	0.91	ACS Energy Lett. 2023, 8, 4531-4539
MnSA/MnACSSCNR	0.1 M KOH	0.90	Small 2023, 2309727
Sb_1/NG(O)	0.1 M KOH	0.86	Angew. Chem. Int. Ed. 2022, 61, e202202200.
Mn SAs/Fe_3C NPs@NPC	0.1 M KOH	0.88	Nano Res. 2022, 15, 7976-7985
Mn-SA@CNSs	0.1 M KOH	0.88	Energy Storage Mater. 2022, 49, 209-218
Ru-SAS/SNC	0.1 M KOH	0.86	J. Am. Chem. Soc. 2022, 144, 2197-2207
Co-CMS	0.1 M KOH	0.83	Adv. Energy Mater. 2022, 12, 2103097
Pd/TiO_2-VO (10 wt%)	0.1 M KOH	0.86	Chem. Eng. J. 2022, 444, 136363.
FeCo-N-HCN	0.1 M KOH	0.86	Adv. Funct. Mater. 2021, 2011289.
Fe SA-NSC-900	0.1 M KOH	0.86	ACS Energy Lett. 2021, 6, 379-386
Co_1-N_3PS/HC	0.1 M KOH	0.92	Angew. Chem. Int. Ed. 2021, 60, 3212-3221
Fe_3Co_7-NC	0.1 M KOH	0.89	Appl. Catal. B: Environ., 2021, 285, 119780
FeN_3OS	0.1 M KOH	0.88	Angew. Chem. Int. Ed., 2021, 60, 25296–25301
$FeMo_2$/NC	0.1 M KOH	0.91	Nano Res., 2022, 15, 4950–4957
